# Detrimental interactions of hypoxia and complement MASP-1 in endothelial cells as a model for atherosclerosis-related diseases

**DOI:** 10.1038/s41598-024-64479-6

**Published:** 2024-06-27

**Authors:** Flóra Demeter, Zsuzsanna Németh, Erika Kajdácsi, György Bihari, József Dobó, Péter Gál, László Cervenak

**Affiliations:** 1 Research Laboratory, Department of Internal Medicine and Haematology, Semmelweis University, Szentkirályi U. 46, Budapest, 1088 Hungary; 2 Research Group for Immunology and Hematology, Semmelweis University—HUN-REN-SU (Office for Supported Research Groups), Budapest, Hungary; 3 Institute of Molecular Life Sciences, HUN-REN Research Centre for Natural Sciences, Hungarian Research Network, Budapest, Hungary

**Keywords:** Cell adhesion, Cell migration, Cell signalling, Cellular imaging, Mechanisms of disease, Complement cascade, Cytokines, Inflammation

## Abstract

Both hypoxia and the complement lectin pathway (CLP) are involved in atherosclerosis and atherosclerosis-related stroke and acute myocardial infarction (AMI). We have previously shown that mannose-binding lectin-associated serine protease-1 (MASP-1), the most abundant enzyme of CLP, induces an inflammatory phenotype of endothelial cells (ECs) by cleaving protease activated receptors (PARs). In the absence of data, we aimed to investigate whether hypoxia and MASP-1 interact at the level of ECs, to better understand their role in atherosclerosis-related diseases. Hypoxia attenuated the wound healing ability of ECs, increased ICAM-1 and decreased ICAM-2 expression and upregulated *PAR2* gene expression. Hypoxia and MASP-1 increased GROα and IL-8 production, and endothelial permeability without potentiating each other’s effects, whereas they cooperatively disrupted vascular network integrity, activated the Ca^2+^, CREB and NFκB signaling pathways, and upregulated the expression of E-selectin, a crucial adhesion molecule in neutrophil homing. VCAM-1 expression was not influenced either by hypoxia, or by MASP-1. In summary, hypoxia potentiates the effect of MASP-1 on ECs, at least partially by increasing PAR expression, resulting in interaction at several levels, which may altogether exacerbate stroke and AMI progression. Our findings suggest that MASP-1 is a potential drug target in the acute phase of atherosclerosis-related diseases.

## Introduction

Atherosclerosis and atherosclerosis-related acute myocardial infarction (AMI) and ischemic stroke are the leading causes of death worldwide. Although their incidence and mortality have declined over the past few decades, cardiovascular diseases still account for an estimated 32% of deaths worldwide, 85% of which are due to AMI and ischemic stroke, according to the World Health Organization. Even with timely and appropriate interventions to restore blood flow, mortality from these diseases is still high. Although reperfusion therapy is essential for patient survival, it can lead to secondary ischemia/reperfusion injury, which may, at least partially, be responsible for mortality^1^. Therefore, there is still a great demand for alternative or supplementary therapeutic options to improve the survival of patients with AMI and stroke.

The complement system is a key component of the innate immune system. Besides acting as a first-line defense mechanism against invading pathogens, it plays a crucial role in the regulation of inflammatory processes and the clearance of apoptotic cells and altered self-structures. It can be activated through three different routes: the classical, the lectin, and the alternative pathways. The initiation of the lectin pathway is mediated by the binding of mannose-binding lectin (MBL), other collectins, or ficolins—pattern recognition molecules (PRMs) of the complement lectin pathway (CLP)—to repeating carbohydrate moieties primarily on pathogen surfaces, but also on apoptotic/necrotic cells, transformed tumor cells, ischemic tissues, and anoxic endothelial cells (ECs)^2–4^. This binding leads to the activation of two mannose-binding lectin associated serine proteases (MASPs: MASP-1 and MASP-2)^2^.

Numerous data demonstrate the involvement of CLP in atherosclerosis and atherosclerosis-related diseases. Lectin pathway activation upon MBL was associated with the vulnerability of atherosclerotic plaques^5^. Ficolin-2 has been found to have a strong predictive value for adverse cardiovascular events^6^. The inhibition or deficiency of MBL provided protection against myocardial and brain ischemia/reperfusion injury in animal models and led to a better prognosis by decreasing the infarct size, tissue injury, C3 deposition, inflammatory gene expression, and neutrophil infiltration^7–11^. In addition, MBL deficiency was associated with a reduction in mortality in patients with acute ST-elevation myocardial infarction undergoing primary percutaneous coronary intervention^12^ and with smaller infarction size and favorable outcome in ischemic stroke patients^13^. In animal models of AMI, C1 inhibitor (C1INH), the predominant natural inhibitor of MASP-1 and -2, provided significant neuro- and cardioprotection by inhibiting complement activation, neutrophil adhesion and infiltration, as well as preserving endothelial function and reducing overall infarct size^14–18^. In addition, protection of myocardial tissue from acute ischemia/reperfusion injury was observed in cases where PAR4, which serves as a receptor for MASP-1, was either inhibited or deficient^19,20^.

At the site of atherosclerotic plaques, damaged macromolecules are often formed on the endothelial cell surface. Among these, acetylated and oxidized macromolecules (e.g., acetylated and oxidized LDL^21^) are important ligands of ficolins. The binding of ficolins leads to the activation of MASP enzymes and, thus, to the initiation of CLP. However, there are still several unanswered questions regarding the exact mechanisms by which CLP might contribute to the development and progression of atherosclerosis and atherosclerosis-related diseases.

MASP-1, the most abundant enzyme of CLP, directly activates ECs through the cleavage of PARs (PAR1, 2 and 4), leading to the activation of the Ca^2+^, nuclear factor kappaB (NF-κB), p38 mitogen-activated protein kinase (MAPK), cAMP-responding element binding protein (CREB) and c-Jun N-terminal kinase (JNK) signaling pathways in cultured human umbilical vein endothelial cells (HUVECs)^22,23^. Upon activation of these pathways, ECs undergo several morphological and functional changes. In HUVECs, MASP-1 induces endothelial permeability via the paracellular route^24^, triggers dose-dependent IL-6 and IL-8 secretion, increases E-selectin and decreases ICAM-2 expression^23,25^ and enhances adhesion between ECs and neutrophils^25^. Taken together, MASP-1 promotes a characteristic proinflammatory phenotype of ECs. In a murine model, an anti-MASP-2 antibody decreased the infarct size and protected the myocardium against ischemia–reperfusion injury^26^. This highlights the importance of MASP-1, as its activation is a prerequisite for the activation of MASP-2^27^. However, to date, there are no direct experimental data on its potential involvement in the pathomechanism of atherosclerosis and atherosclerosis-related diseases.

ECs form the inner cellular lining of blood and lymphatic vessels, representing an important interface between the vasculature and the underlying tissues. Postcapillary venule ECs are involved in leukocyte trafficking by mediating initial attachment, rolling, firm adhesion, and transmigration through the expression of adhesion molecules, such as E- and P-selectin, vascular cell adhesion molecule 1 (VCAM-1), and intercellular cell adhesion molecule 1 and 2 (ICAM-1 and ICAM-2)^28^.

Hypoxia and hypoxia/reoxygenation (H/R) are direct, central components of the pathophysiology of ischemic stroke and AMI. H/R-induced oxidative stress produces oxidized sugar moieties that act as ligands for ficolins, PRMs of CLP. Moreover, it has a direct effect on ECs via reactive oxygen species (ROS) generation.

CoCl_2_ is a well-known, commonly used hypoxia-mimetic agent that can create chemical hypoxia^29^. It acts via strongly stabilizing hypoxia-inducible factors 1α and 2α under normoxic conditions, mainly by inhibiting the hidroxylating activity of prolyl-hydroxylases by displacing Fe^2+^ by Co^2+^. As the HIF-stabilizing effect is maintained for several hours, the hypoxic milieu remains unchanged during treatments or other manipulations requiring the opening of the culture plate/flask. Several similarities between low O_2_ and CoCl_2_-induced hypoxia have been reported, making CoCl_2_ a reliable alternative hypoxia model^29^.

Both hypoxia and the activation of CLP have been shown to play an important role in atherosclerosis and atherosclerosis-related diseases; however, their combined effect has never been studied before. In this article, we demonstrate the extensive interaction of hypoxia and MASP-1 on ECs at the level of signaling pathways, adhesion molecules and vascular network integrity for the first time.

## Results

To study the interaction of hypoxia and MASP-1, we used two experimental setups: pre-treatment with CoCl_2_ followed by MASP-1 treatment or simultaneous treatment with CoCl_2_ and MASP-1. Figures with a colored background indicate CoCl_2_ pre-treatment, and those with a white background the simultaneous application of the agents. Before conducting our experiments, we validated the CoCl_2_ hypoxia model by demonstrating its time- and dose-dependent impact on HIF-1α nuclear translocation while also confirming the absence of any cytotoxic effects on ECs (see Supplementary Fig. 1 and 2 online). Our previous studies^22–25,30,31^ suggest that a MASP-1 concentration of 2 µM is regarded as optimal for most experimental setups. However, as in our previous study^32^, we deliberately used a suboptimal dose of 0.6 µM, to sensitively detect any potential interactions with hypoxia.

### Adhesion molecule expression

**Figure 1:**
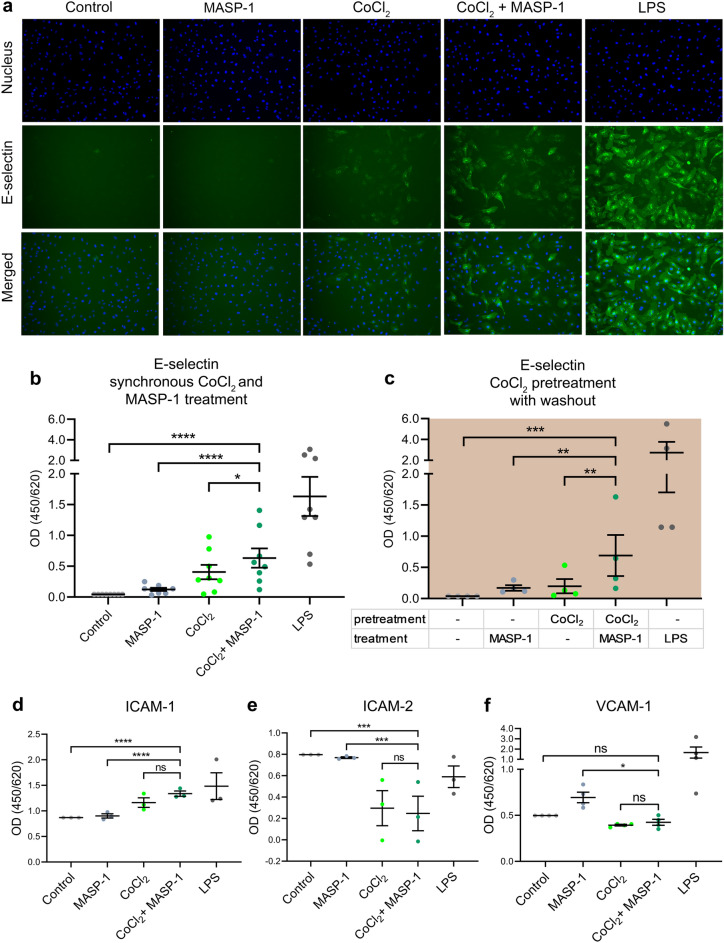
The effect of CoCl_2_ and rMASP-1 on adhesion molecule expression HUVECs were treated with 400 μM of CoCl_2_ or 0.6 μM of rMASP-1 or both for 6h (**a**, **b**) or 24h (**d**, **e**, **f**). Alternatively, cells were pretreated with 400 μM of CoCl_2_ for 2 h and received 6h of 0.6 μM of rMASP-1 treatment after washing the CoCl_2_ out (**c**). We used 100 ng/ml of LPS as a positive control. To visualize E-selectin expression with fluorescence microscopy, cells were stained with mouse anti-human E-selectin followed by Alexa Fluor568-conjugated goat anti-mouse IgG (for better visualization, fluorescence was presented in a green color) and Hoechst 33,342 (blue) as nuclear staining for 1h (**a**). To enumerate the difference, cellular ELISAs were also performed. Cells were stained with mouse anti-human E-selectin, ICAM-1, ICAM-2, or VCAM-1 antibodies (1:500) for 1h, followed by HRP-conjugated goat anti-mouse antibody and 3,3’,5,5’-Tetra Methyl Benzidine (TMB) for 1h (**b-f**). n = 5–24 (3–8 biological replicates, 1–3 technical replicates). Graphs represent individual values and mean ± SEM. Nested one-way ANOVA with a Sidak multiple comparison post-test. P values are as follows: ****: < 0.0001, ns: nonsignificant (**b**): *: 0.0162, (**c**): **: 0.0044 (MASP-1 vs CoCl_2_ + MASP-1), 0.0074 (CoCl_2_ vs CoCl_2_ + MASP-1), ***: 0.0003, (**e**): ***: 0.0001 (Control vs CoCl_2_ + MASP-1), 0.0003 (MASP-1 vs CoCl_2_ + MASP-1), (**f**): *: 0.0444.

EC surface adhesion molecules facilitate the binding of various cells to the endothelium. We previously demonstrated that MASP-1 enhances neutrophil adhesion by increasing E-selectin but not ICAM-1 or VCAM-1 expression^25^. Hypoxia was also shown to promote EC-neutrophil interactions, among others, through increased adhesion molecule expression, such as ICAM-1^33^. Therefore, we first studied the combined effect of the two agents on the expression of various adhesion molecules. HUVECs were treated with CoCl_2_, rMASP-1 or both of them for 6 (E-selectin) or 24 h (ICAM-1, ICAM-2, VCAM-1). We showed that E-selectin, an adhesion molecule crucial for neutrophil homing, was cooperatively upregulated by CoCl_2_ and rMASP-1 (Fig. 1a and b), despite suboptimal dose of rMASP-1 alone having only a slight, nonsignificant effect. Given the observed interaction, we pretreated cells with CoCl_2_ for 2h, then added rMASP-1 after washing the CoCl_2_ out to investigate whether a shorter, temporary hypoxic exposure could similarly sensitize endothelial cells to the effect of rMASP-1. CoCl_2_ pretreatment increased E-selectin expression in response to rMASP-1 (Fig. 1c). CoCl_2_, but not rMASP-1, increased ICAM-1 and decreased ICAM-2 expression (Fig. 1d and e), resulting in a bias in their expression difference (see Supplementary Fig. 3 online), regardless of the presence of rMASP-1. Neither CoCl_2_, nor rMASP-1 had an effect on VCAM-1 expression (Fig. 1f). LPS was used as a positive control in all experiments.

### Cytokine production

**Figure 2:**
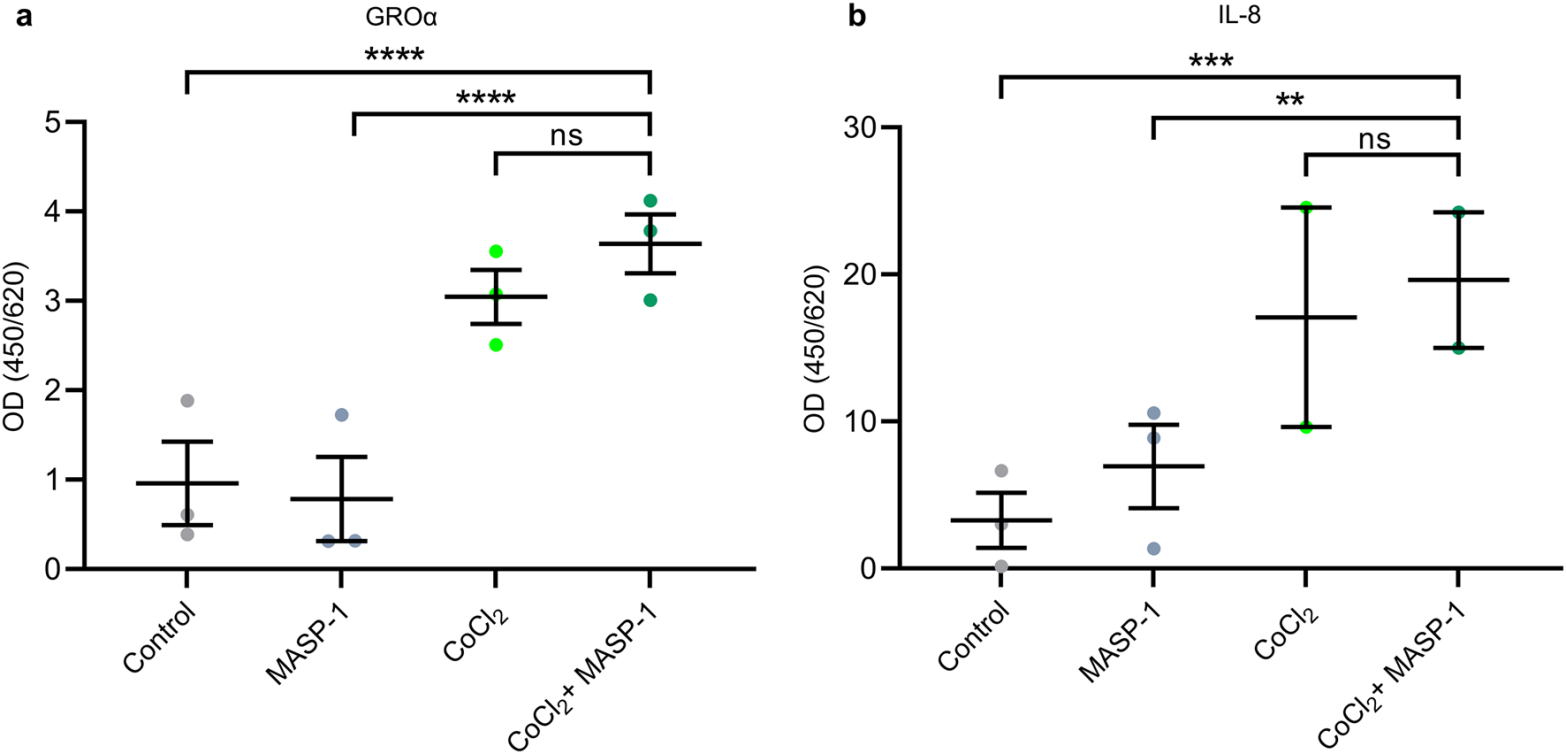
The effect of CoCl_2_ and rMASP-1 on cytokine production HUVECs were treated with 400 μM of CoCl_2_ or 0.6 μM of rMASP-1 or both for 24 h. GROα (**a**) and IL-8 (**b**) production was measured from the supernatants by sandwich ELISA kits according to the manufacturer’s protocol. n = 5–14 (3 biological replicates, 1–5 technical replicates). Graphs represent individual values and mean ± SEM. Nested one-way ANOVA with a Sidak multiple comparison post-test. P values are as follows: **: 0.0053, ***: 0.0003, ****: < 0.0001, ns: nonsignificant.

Proinflammatory cytokines, produced by ECs, can act as chemotactic factors for different types of leukocytes. As CoCl_2_ and rMASP-1 cooperatively increased the expression of E-selectin, an adhesion molecule important in neutrophil homing, we investigated whether the two agents could alter the production of two neutrophil chemotactic cytokines, GROα and IL-8. HUVECs were treated with CoCl_2_, rMASP-1 or both for 24 h, then the level of secreted cytokines was determined from the supernatants. We found that CoCl_2_ increased both GROα and IL-8 production, whereas suboptimal rMASP-1 treatment alone had no significant effect on either of them. In combination with CoCl_2,_ rMASP-1 increased GROα and IL-8 production slightly more than CoCl_2_ alone, although these correlations were not statistically significant (Fig. 2).

### Endothelial permeability

**Figure 3:**
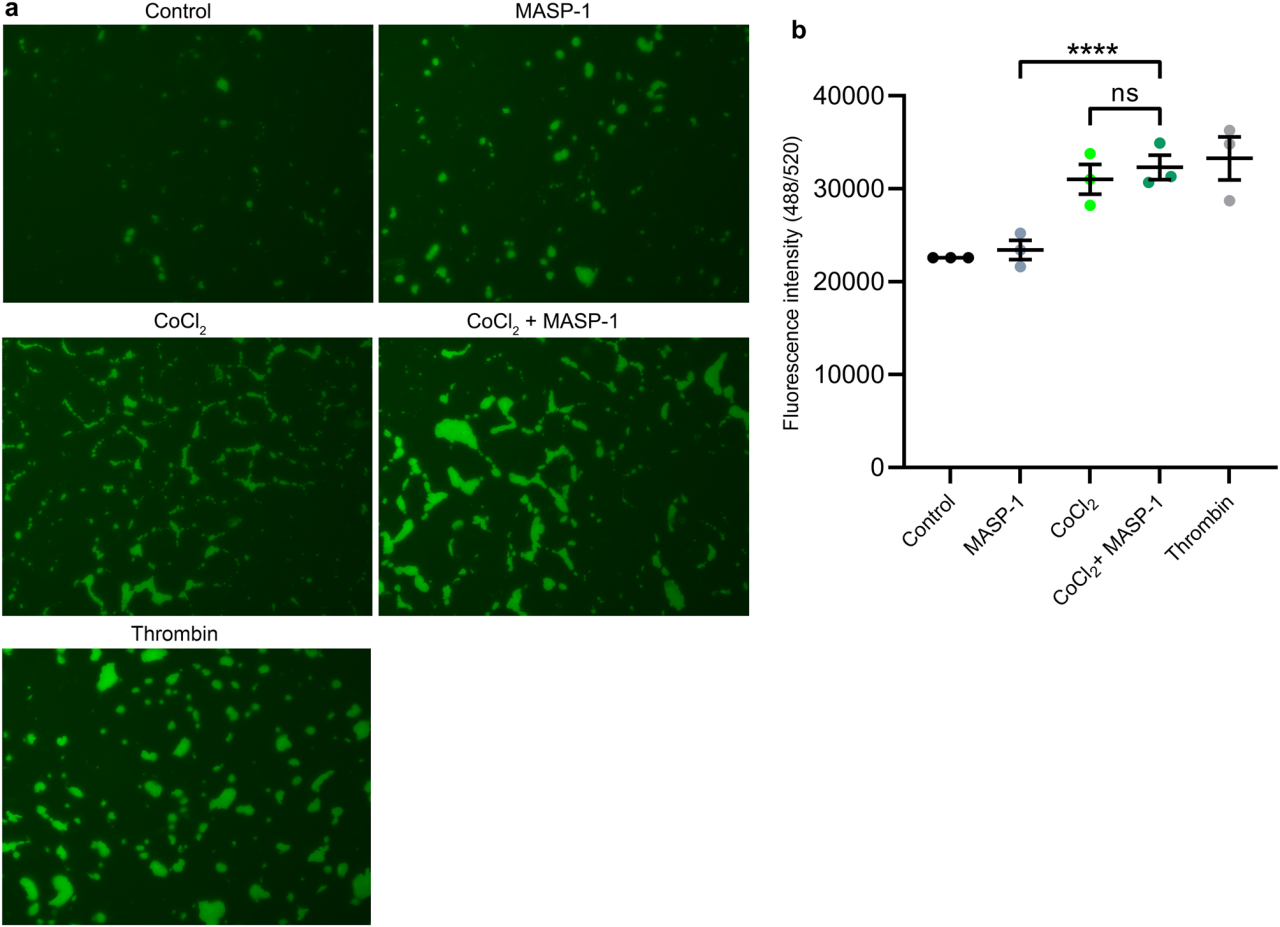
The effect of CoCl_2_ and rMASP-1 on endothelial permeability HUVECs were seeded onto 96-well plates, pre-coated with 250 μg/ml biotinylated gelatin. We used 1 U/ml of thrombin as a positive control. After treatment with 400 μM of CoCl_2_ for 24 h and/or 0.6 μM of rMASP-1 for 20 min, 2 μg/ml Streptavidin-Alexa488 (green) was applied to each well for 2 min, and cells were fixed in 1% paraformaldehyde-PBS. An Olympus IX-81 fluorescence microscope and an Olympus XM-10 camera were used to take pictures of each well. Panel (**a**) represents one set of photos from three independent experiments. To determine the size of the stained area, the plates were read by a fluorescent plate reader. Panel (**b**) shows the fluorescence values normalized to controls. n = 9 (3 biological replicates, 3 technical replicates). The graph represents individual values and mean ± SEM. Nested one-way ANOVA with a Sidak multiple comparison post-test. P values are as follows: ****: < 0.0001, ns: nonsignificant.

Considering that the regulation of vascular permeability is a major function of ECs, we conducted a permeability test. We used a modified version of the recently developed XPerT method. Cells were treated with rMASP-1 for 20 min or CoCl_2_ for 24 h with or without rMASP-1 in the last 20 min of the treatment. We present a set of representative fluorescence microscopic photos (Fig. 3a) and quantified the results using a less sensitive but more objective fluorescent plate reader, with the fluorescent values plotted in Fig. 3b. CoCl_2_, but not rMASP-1, increased the endothelial permeability, with only a statistically nonsignificant trend towards interaction observed. The change in permeability triggered by the two agents together was comparable to the effect of thrombin, used as a positive control.

### Wound healing

**Figure 4:**
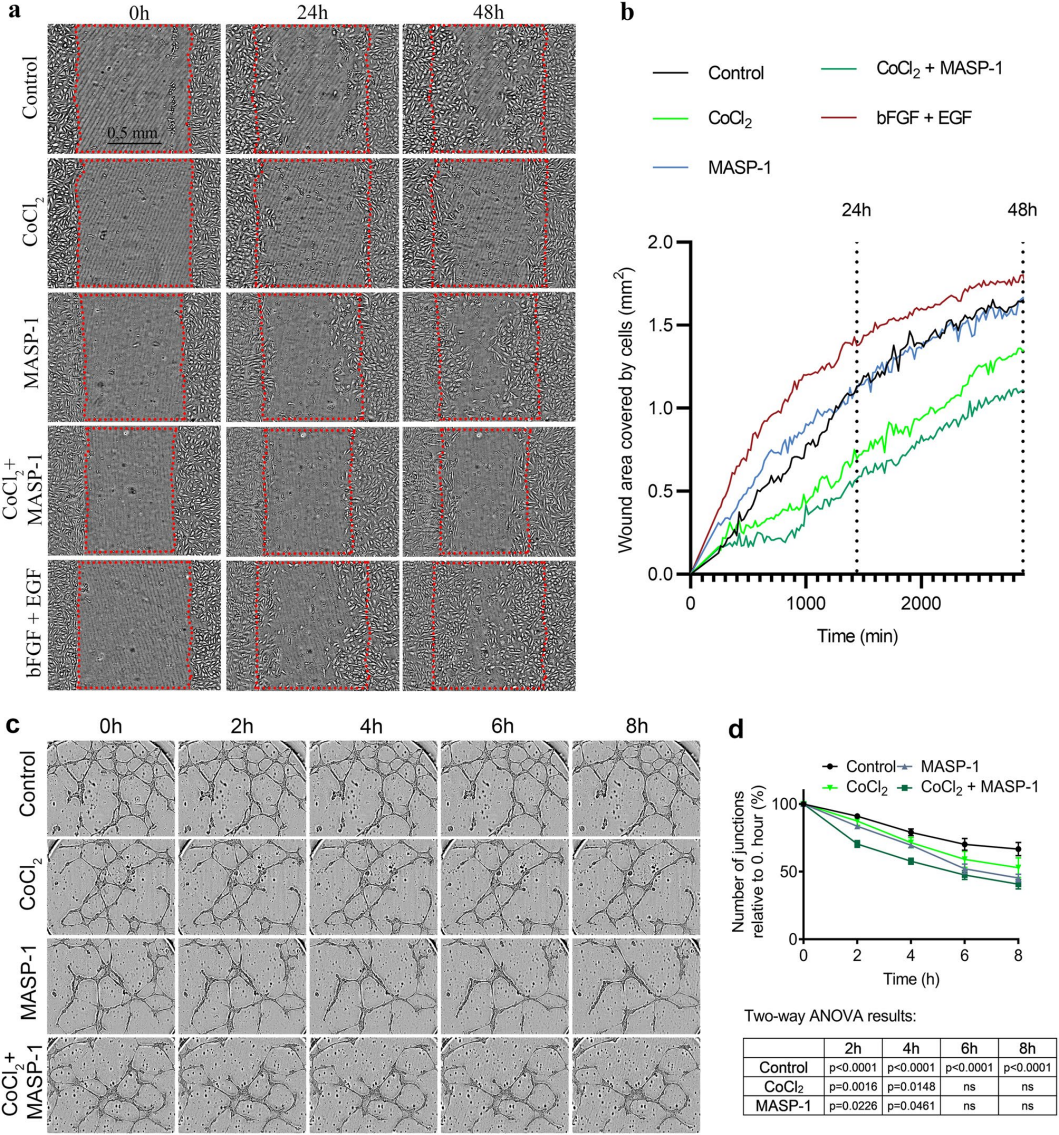
The effect of CoCl_2_ and rMASP-1 on wound healing and capillary network integrity HUVECs were cultured in 96-well plates until reaching full confluency, when a scratch was created on the cell layer (**a**, **b**). After a single rinse with MCDB medium, cells were treated with 0.6 μM of rMASP-1, 400 μM of CoCl_2_, or both. A combination of 5 ng/ml of bFGF and 10 ng/ml of EGF was used as a positive control. The Olympus CM30 Incubation Monitoring System was used to capture images at 20 min intervals, allowing for the observation and analysis of cell confluency. Panel (**a**) represents one set of photos from three independent biological replicates. The scale bar in the first picture is consistent across all 15 images, measuring 0.5 mm. Curves and input levels were adjusted for better visualization, while calculations were performed based on the original, unaltered images (Supplementary Video 1). Panel (**b**) shows the cell coverage of the wound area in mm^2^. n = 4–6 (2–3 biological replicates, 2 technical replicates). Alternatively, HUVECs were seeded onto Matrigel^™^-coated 15-well Angiogenesis µ-Slides at 120% confluency (**c**, **d**). After an 18 h period of tube formation, cells were treated with either 0.6 μM of rMASP-1, 400 μM of CoCl_2_, or a combination of both. Images were obtained with an Olympus CM30 Incubation Monitoring System. Panel (**c**) represents one set of photos from three independent experiments. (For further details see Supplementary Video 2.) The quantification of branching points was performed using Image J software. The values on the time kinetic curves are relative to 0 h (**d**). n = 5–7 (2–3 biological replicates, 2–3 technical replicates). The table below the graph presents the significance of the combined CoCl_2_ and MASP-1 treatment in comparison to both the control group and the individual treatments at each time point. Two-way ANOVA with a Tukey’s multiple comparison post-test. *ns* nonsignificant.

ECs play a pivotal role in wound healing at sites of vascular injury, especially during thrombotic events. Subsequently, we investigated the impact of hypoxia and MASP-1 on their cell migration and wound healing ability. Confluent HUVEC monolayers were scratched, then treated with CoCl_2_, rMASP-1 or both. rMASP-1 alone had no effect, whereas CoCl_2_ with or without rMASP-1 attenuated the migratory and wound healing ability of HUVECs during the 30h observation period. The combined effects of the two agents were slightly stronger compared to the effect of CoCl_2_ alone, although this correlation was not statistically significant (Fig. 4a and b**,** see Supplementary Video 1 online).

### Capillary network integrity

ECs are responsible for maintaining vascular integrity; therefore, we studied how MASP-1 and CoCl_2_ affect this crucial function. After 18 h of tube formation on Matrigel-coated µ-Slides, the established capillary networks of HUVECs were treated with CoCl_2_, rMASP-1, or a combination of both. We observed that rMASP-1 and CoCl_2_ cooperatively disrupted the vascular network integrity at 2 and 4 h, whereas at later times, their combined effect resembled that of rMASP-1 alone (Fig. 4c and d**,** see Supplementary Video 2 online).

### Signaling pathways

The observed interaction of hypoxia and MASP-1 at the level of adhesion molecules and capillary network integrity measurements prompted us to investigate their effects on various signaling pathways to better understand the underlying mechanisms by which they potentially interact.

#### Ca^2+^ mobilization

**Figure 5:**
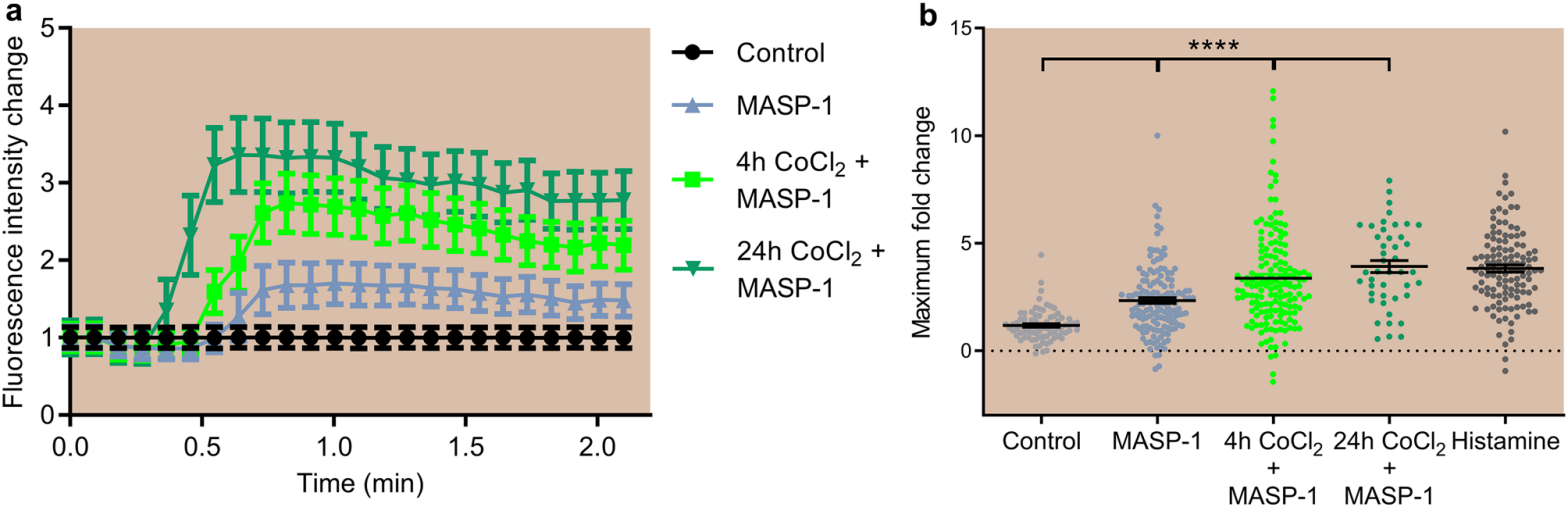
CoCl_2_ induced Ca^2+^ mobilization in response to rMASP-1 Part of HUVECs was pretreated with 400 μM of CoCl_2_ for 4 h or 24 h. After loading the cells with Fluo-4-AM, we performed fluorescence microscopy and determined baseline fluorescence by taking three photos. Then, 0.6 μM of rMASP-1 treatment was added and sequential images were obtained every 5 s for 2 min. Changes in fluorescence intensity were calculated based on the analysis of at least 20 cells per image, using CellP software. Panel (**a**) is a representative kinetic curve of three independent experiments. Panel (**b**) shows the maximum values of the kinetic curves (maximum fold change). We used 50 μM of Histamine as a positive control. n = 44–160 (3 biological replicates, 14–40 cells). The graph represents individual values and mean ± SEM. One-way ANOVA with a post-test for linear trends. P values are as follows: ****: < 0.0001.

In signaling pathways, Ca^2+^ acts as a secondary messenger for numerous receptors, such as Protease Activated Receptors (PARs), members of the G protein coupled receptor family. By cleaving PARs, rMASP-1 initiates several signaling pathways, where Ca^2+^ mobilization is an early, easily detectable step; therefore, we first investigated the interaction with hypoxia here. We pretreated cells with CoCl_2_ for 4 h or 24 h, then loaded them with Fluo-4-AM, a Ca^2+^-sensitive fluorescent dye, before adding rMASP-1 treatment. We found that both 4 and 24h of CoCl_2_ pretreatment enhanced the maximal Ca^2+^ mobilization in response to rMASP-1 (Fig. 5). This effect was comparable to the effect of the well-known histamine, used as a positive control.

#### NFKB nuclear translocation

**Figure 6:**
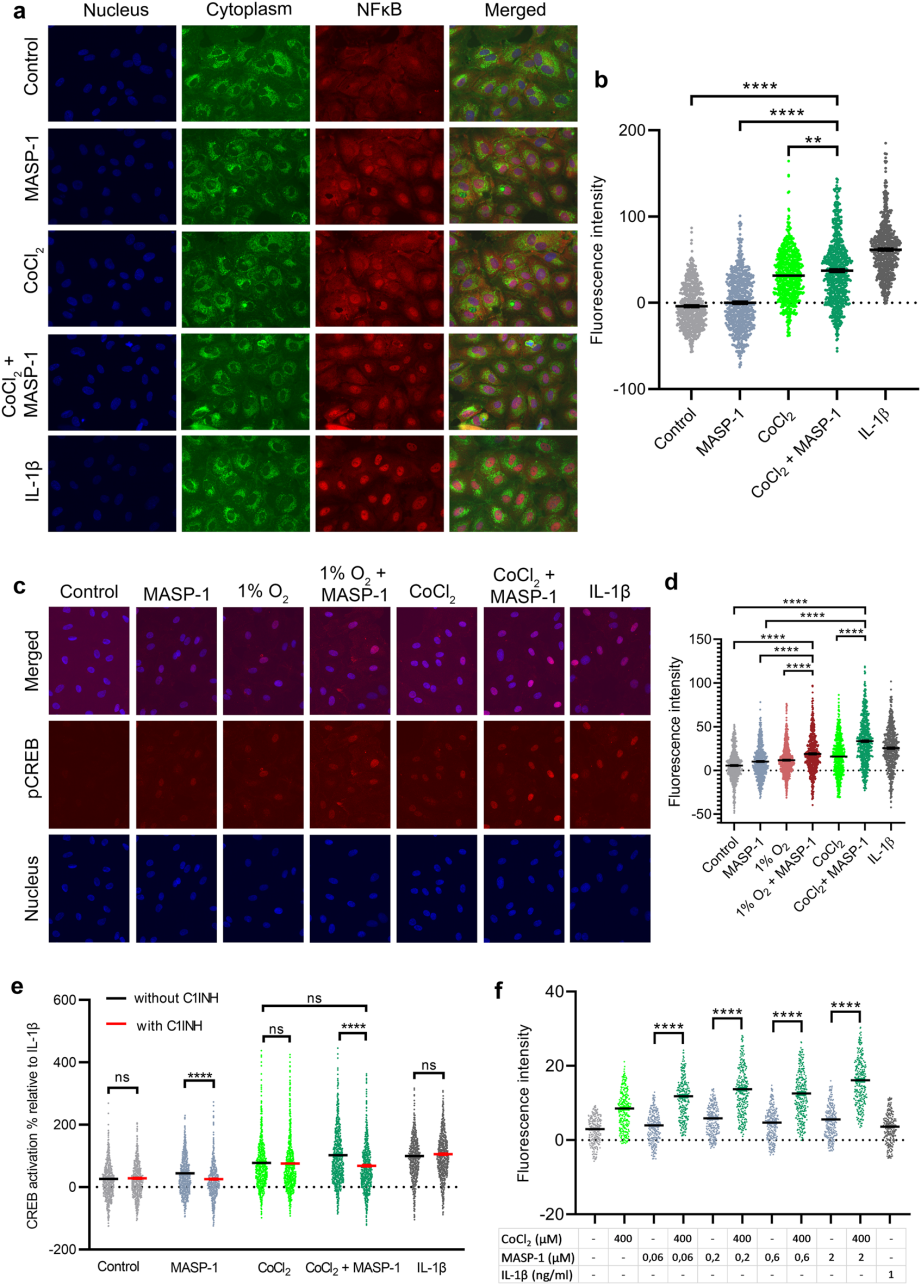
The effect of CoCl_2_/ 1% O_2_, rMASP-1 and C1INH on NFκB and CREB signaling pathway For the NFκB experiments, HUVECs were treated with 0.6 μM of rMASP-1 for 1h or 400 μM of CoCl_2_ for 2 h with or without rMASP-1 in the last 1h (**a**, **b**). For the CREB experiments, HUVECs were treated with 0.6 μM of rMASP-1, 400 μM of CoCl_2_, or both for 2 h with or without 6 μM of C1INH (**c**, **d**, **e**). Another plate of HUVECs was placed in a hypoxic (1% O_2_) incubator with or without 0.6 μM rMASP-1 treatment for 2 h (**c**, **d**). In another experimental setup, HUAECs were treated with varying concentrations of rMASP-1 (0.06 μM, 0.2 μM, 0.6 μM, 2 μM), 400 μM of CoCl_2_, or the combination of each dose of rMASP-1 with CoCl_2_ for 2 h (**f**). We used 1 ng/ml of IL-1β as a positive control. After Methanol-acetone fixation, cells were stained with rabbit-anti-human phospho-CREB (1:200) or rabbit-anti-human NFκB (1:250), followed by Alexa Fluor568-conjugated goat anti-rabbit (1:500, red) IgG and Hoechst 33,342 (1:50,000, blue). Additionally, wheat germ agglutinin (1:200, green) was applied for the NFκB experiments. An Olympus IX-81 fluorescence microscope and an Olympus XM-10 camera were used to take photos (**a**, **c**). Panel (**a**) and (**c**) represents one set of photos from three independent experiments. Photos were analyzed with the CellP software (Olympus). For the NFκB experiments, the difference between the cytoplasmic and nuclear mean red fluorescence was calculated (**b**). n = 720 (3 biological replicates, 240 cells). For the CREB experiments, the nuclear mean red fluorescence (**d**, **f**) or the percentage of the nuclear mean red fluorescence relative to IL-1β (**e**) was calculated after subtracting the cell-free background fluorescence values. n = 630–1200 (HUVEC: 3 biological replicates, HUAEC: 1 biological replicate, 210–400 nuclei). Graphs represent individual values and mean ± SEM. One-way ANOVA (**b**, **d**, **e**) or two-way ANOVA (**f**) with a Sidak multiple comparison post-test. P values are as follows: **: 0.0034, ****: < 0.0001, ns: nonsignificant. One-way ANOVA with a post-test for linear trends (**f**) for both the rMASP-1 and the CoCl_2_ + rMASP-1 dose–response analyses was performed (p values < 0.0001); however, they are not depicted on the figure for enhanced clarity.

Our research group has previously demonstrated that optimal MASP-1 is able to increase NFκB nuclear translocation^22^. It has also been described that hypoxia is able to exert the same effect^34^. HUVECs were treated with rMASP-1 for 1 h or CoCl_2_ for 2 h with or without rMASP-1 in the last 1 h of the treatment. Suboptimal concentrations of rMASP-1 alone failed, whereas CoCl_2_ was able to induce NFκB nuclear translocation. On average, the combined effect of CoCl_2_ and rMASP-1 was stronger than the agents alone. However, there was a high variance between independent cell lines, ranging from a weaker to a stronger reaction to the combined treatment than to the agents alone (Fig. 6a and b).

#### CREB phosphorylation

Similarly to the NFκB pathway, activation of the CREB pathway both by MASP-1^23^ and hypoxia^35^ is also well documented. To study the phosphorylation of CREB, HUVECs were treated with CoCl_2_, rMASP-1, or both for 2 h. In addition, another plate of HUVECs was placed in a hypoxic (1% O_2_) incubator with or without rMASP-1 treatment for 2 h, to verify our results with the CoCl_2_ hypoxia model. Both 1% O_2_ hypoxia, CoCl_2_ and rMASP-1 increased the pCREB concentration in the nuclei. The combined effect of CoCl_2_ and rMASP-1 was even greater than their effects alone, and comparable to that of IL-1β, used as a positive control. Moreover, 1% O_2_ hypoxia and rMASP-1 cooperatively activated the CREB signaling pathway, similarly as CoCl_2_ and rMASP-1 (Fig. 6c and d).

In another experimental setup, HUVECs were treated with CoCl_2_, rMASP-1, or both for 2 h with or without C1INH, a natural inhibitor of rMASP-1. C1INH inhibited the hypoxia-enhancing effect of MASP-1 on CREB phosphorylation, whereas it had no impact on control cells or on the effect of IL-1β or CoCl_2_ alone (Fig. 6e).

To generalize the effect of CoCl_2_ and rMASP-1 to other endothelial cell types, we replicated the CREB experiment using a HUAEC (Human Umbilical Artery Endothelial Cell) line. Consistent with our findings in HUVECs, CoCl_2_ and rMASP-1 cooperatively activated the CREB signaling pathway in HUAECs (Fig. 6f). Additionally, we observed a slight dose-dependent effect of rMASP-1 in potentiating the effect of hypoxia on CREB phosphorylation both in HUVECs (see Supplementary Fig. 4 online) and HUAEC (Fig. 6f).

### PAR1, 2 and 4 gene expression

Endothelial cell surface PARs are the major receptors of rMASP-1. Their cleavage by rMASP-1 leads to the initiation of several signaling pathways and ultimately to the activation of ECs. As there are no data on whether hypoxia can affect endothelial PAR expression, we investigated its potential effect on the level of gene expression.

**Figure 7:**
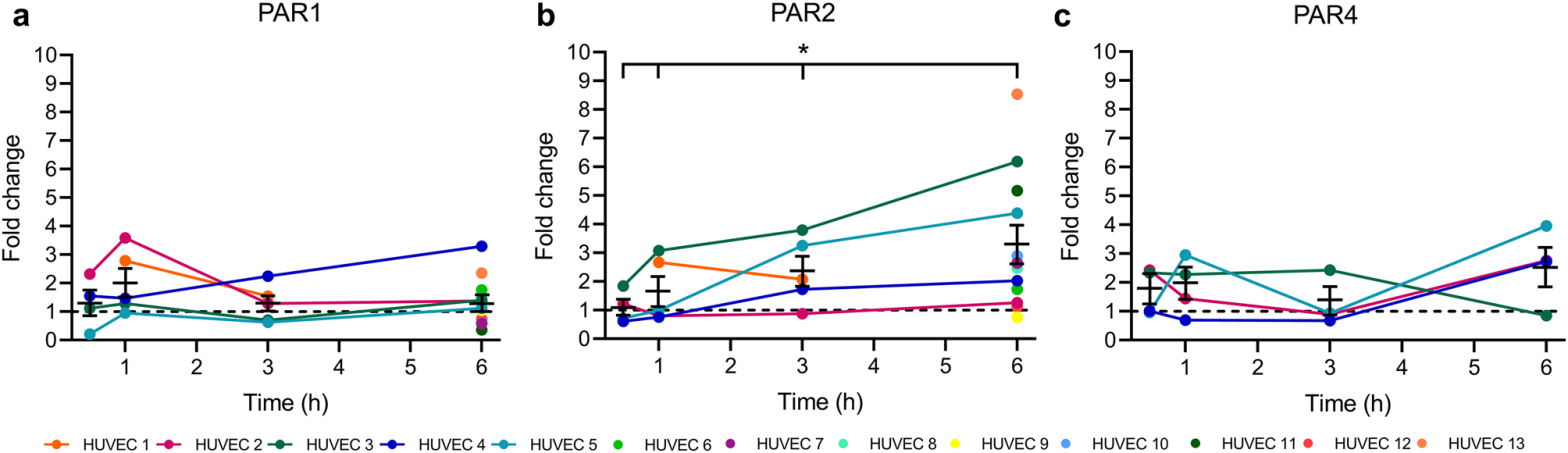
The effect of CoCl_2_ on Par1, 2, and 4 gene expression HUVECs were treated with 400 μM of CoCl_2_ for 30 min, 1 h, 3 h or 6 h. After mRNA isolation and mRNA-cDNA reverse-transcription, quantitative real-time PCR was applied. n = 8–24 (4–12 biological replicates, 2 technical replicates). Graphs represent individual values and mean ± SEM, and show the kinetics of four individual HUVEC lines (**a**, **b**, **c**). Nested one-way ANOVA with a post-test for linear trends. *p = 0.0348.

HUVECs were treated with CoCl_2_ for 30 min, 1h, 3h or 6h. After mRNA isolation, quantitative real-time PCR was applied to study PAR1, 2 and 4 gene expression. We found that CoCl_2_ increased *PAR2* gene expression in a time-dependent manner, with a maximum effect at 6h (Fig. 7b and e). The results were inconclusive for *PAR1* (Fig. 7a and d) and *PAR4* (Fig. 7c and f).

## Discussion

In the present study, we found that hypoxia and MASP-1 cooperatively increased endothelial E-selectin expression, disruption of endothelial network on Matrigel, Ca^2+^ mobilization, CREB phosphorylation, and NFκB nuclear translocation. In addition, hypoxia upregulated the expression of the MASP-1 receptor PAR2. Our observations are the first to demonstrate that hypoxia and MASP-1, the most abundant enzyme of the complement lectin pathway, can enhance each other’s effects.

Complement lectin pathway plays an important role in atherosclerosis-related acute thrombotic events^7,9,13,17,36^, in which hypoxia and hypoxia/reoxygenation are also direct pathogenic components. Despite the fact that, MASP-1, a thrombin-like protease with a plasma concentration of 143 nM^37^, is the most abundant enzyme of CLP, there has been very limited research on its potential effects in stroke and AMI. Our research group has previously demonstrated that MASP-1 induces a proinflammatory phenotype of endothelial cells, which is also a common consequence of hypoxia and hypoxia/reoxygenation. In addition, *Noguchi *et al*.* have recently demonstrated that thrombin exacerbates hypoxia/reoxygenation injury of sinusoidal endothelial cells and hepatocytes in a mouse model^38^. This interaction is very similar to the one observed in our experiments between MASP-1 and hypoxia, which supports our hypothesis that the two agents act together.

After the cleavage of PARs^22,23^, MASP-1-induced activation of the Ca^2+^, NFκB, p38-MAPK, JNK, and CREB signaling pathways triggers changes in cell surface adhesion molecule expression. The pattern of adhesion molecules, as well as cytokines, expressed by the endothelium, determines the migration of different leukocyte subsets. Our research group previously showed that MASP-1 upregulates the expression of E-selectin, a crucial adhesion molecule for neutrophil homing^25^. On the other hand, there are contradictory data on whether hypoxia can affect endothelial E-selectin expression. *Kim *et al*.*^39^ and *Song *et al*.*^40^ reported increased E-selectin expression following hypoxia in HUVECs and in pulmonary artery ECs, respectively, whereas *Maurus *et al*.*^37^ and *Liang *et al.^41^ found no alteration in human aortic ECs and in HUVECs, respectively. These contradictory findings may stem from EC heterogeneity and variations in experimental setups and cell culture conditions. In our experiments, E-selectin expression was upregulated by hypoxia, but not the suboptimal dose of MASP-1. VCAM-1 is an adhesion molecule predominantly involved in the transmigration of monocytes and activated T lymphocytes. We found that neither MASP-1, nor hypoxia had an effect on VCAM-1 protein expression, which is in line with our previous findings^25^. In our experiments, not only prolonged hypoxia, but even a short, transient hypoxic environment could interact with MASP-1 in inducing E-selectin expression, whereas VCAM-1 expression was not influenced by either agent. The changes observed in the pattern of adhesion molecules facilitate neutrophil homing, as opposed to that of T lymphocytes and monocytes. Our finding that hypoxia can enhance the effect of a proinflammatory stimulus (e.g., MASP-1) on E-selectin expression is not unprecedented, as it was already observed for LPS and TNFα^42^. ICAM-1 is an adhesion molecule involved in the transmigration of all types of leukocytes, whereas ICAM-2 may function as a decoy molecule. By upregulating the expression of ICAM-1 and downregulating that of ICAM-2, hypoxia promotes cell transmigration in general, whereas MASP-1 does not enhance this effect further.

Hypoxia can also enhance the production of proinflammatory cytokines, such as IL-6^43^, IL-8^44,45^, and IL-1α^46^. Furthermore, HIF-1α was proven to upregulate GROα production by inducing miR-19a expression^47^. As our finding suggests that hypoxia and MASP-1 together promote the recruitment of neutrophils at the level of adhesion molecules, we investigated their combined effect on the production of IL-8 and GROα, two important neutrophil chemotactic cytokines. We found that hypoxia, in line with previous findings^44,47^, enhanced the endothelial production of both IL-8 and GROα, whereas the suboptimal dose of MASP-1 alone failed to exert the same effects. Although it was not significant statistically, the two agents together had a slightly greater effect than hypoxia alone. Together with the increased E-selectin expression, this trend toward interaction can contribute to the proinflammatory response and favor the recruitment of neutrophils.

The regulation of permeability during inflammation is a major function of the endothelium. Both MASP-1^24^, and hypoxia^48^, can increase the vascular permeability, facilitating the extravasation of soluble immune mediators and the transmigration of immune cells. MASP-1-induced changes in permeability are mediated by intracellular Ca^2+^ mobilization, MLC phosphorylation, and cytoskeletal actin rearrangement^24^, whereas hypoxia acts by lowering the cellular cAMP levels^48^. In our experiments, 24 h of hypoxia largely increased the endothelial permeability, and MASP-1 failed to significantly enhance its effect further. However, as with IL-8 or GROα, a trend toward interaction was observed, although statistically nonsignificant, which, together with other changes triggered by the two agents, might contribute to the inflammatory response.

The integrity of the vascular wall is vital for normal vascular function, which gets compromised upon injury, prompting ECs to migrate and repair the damaged surface, a process known as wound healing^49,50^. In AMI or stroke, factors such as reactive oxygen species, inflammation, and ischemia damage microvascular endothelial surfaces, which aggravates disease progression^18,26^. We found that hypoxia reduces the migratory and wound healing abilities of HUVECs, with MASP-1 slightly further exacerbating this effect, delaying endothelial repair and impeding the healing of the damaged endothelial surface.

An intricate capillary network is essential for adequate supply of oxygen and nutrients to tissues. During acute thrombotic events, such as AMI or stroke, the hypoxic area behind the occluded artery relies on the collateral capillary system for oxygen^50^. Our findings reveal a cooperative disruption of capillary network integrity induced by the combination of hypoxia and MASP-1 in the first 4 h, raising the possibility that early administration of a MASP-1 inhibitor has the potential to improve disease prognosis.

Ca^2+^ is a second messenger in several signal transduction pathways, for example, at G protein-coupled receptors such as PARs. We have previously demonstrated that MASP-1 induces a Ca^2+^ signal in HUVECs via cleaving the PAR receptors^22^, which, together with other signaling pathways (e.g., JNK, CREB, NFκB, p38-MAPK, MLC), leads to the alteration of endothelial permeability, cell surface adhesion molecule expression, and cytokine production. Here we showed for the first time that hypoxia pretreatment can enhance Ca^2+^ mobilization in response to MASP-1 in a time-dependent manner. This raises the possibility that in a setting of acute hypoxia, direct endothelial effects of MASP-1 are exacerbated.

According to the Signaling Pathways Project (SPP) knowledgebase, of the adhesion molecules and cytokines studied, only IL-8, ICAM-1, and ICAM-2 have HIF-1α binding sites in their promoter regions. This implies that hypoxia-induced upregulation of these proinflammatory molecules and interaction with MASP-1 take place through other intracellular routes. All of the adhesion molecules and cytokines we studied carry CREB and NFκB transcription factor-binding sites in their promoter regions. Moreover, we previously demonstrated that MASP-1 is able to activate the CREB and NFκB signaling pathways^22,23^. The ability of hypoxia to exert the same effects has also been described^34,35^. In this study, we demonstrate for the first time that hypoxia and MASP-1 cooperatively activate the CREB and NFκB signaling pathways. This, together with the fact that hypoxia enhances MASP-1-induced Ca^2+^ mobilization, can potentially underlie their interaction at the level of adhesion molecules and capillary network integrity.

In our experiments, a consistent concentration of 0.6 μM of MASP-1 was employed, representing four times the plasma concentration. However, the dose–response analysis part of the CREB experiment revealed that even lower concentrations of MASP-1 elicited noticeable effect. Additionally, it is worth noting that the CLP pattern recognition molecules can anchor MASP-1 to the endothelial cell surface^3,51^, potentially leading to local concentrations higher than those in plasma, supporting the relevance of the MASP-1 dose used in our experiments.

PARs are the main receptors through which MASP-1 exerts its effects on endothelial cells^22^. MASP-1-mediated cleavage of PAR1,2 and 4 results in the initiation of distinct signaling pathways, including the Ca^2+^, CREB, and NFκB routes. *Zhang *et al. have recently shown that myocardial ischemia–reperfusion injury reduced the amount of PAR1, whereas enhanced that of PAR4 in the murine myocardial tissue^18^. Here, we demonstrate for the first time that hypoxia upregulates the gene expression of *PAR2* in ECs in a time-dependent manner, with the greatest effect at 6 h. This implies that hypoxia potentiates the effect of MASP-1 on endothelial cells, at least partly by increasing the expression of PAR2, a well-known receptor of MASP-1 but not excluding the possible role of PAR-1 and PAR-4, as hypoxia-induced expression of PAR1 and 4 occurred at different time points in different HUVEC lines. In addition to cooperating at the level of signaling pathways, this finding provides an additional explanation for how hypoxia and MASP-1 can interact.

A limitation of our study is the use of CoCl_2_ instead of low oxygen-induced hypoxia^29^, the most natural and optimal hypoxia model. On the one hand, CoCl_2_ is a widely used chemical hypoxia-mimetic agent^29^ that has been shown to induce HIF-1α nuclear translocation in various cell lines, including MCF-7 cells^52^ and HeLa/HepG2 cells^53^ as well as in HUVECs^54–56^, as confirmed by our own experiments (see Supplementary Fig. 1 online). All these suggest that CoCl_2_ mimics the effects of low oxygen-induced hypoxia on the HIF-1α signaling pathway; however, it remains uncertain whether it fully replicates all the effects associated with other signaling pathways. On the other hand, the use of low oxygen-induced hypoxia also has its own limitations, notably the reintroduction of O_2_ into the hypoxic chamber at each opening, resulting in an uncertain O_2_ concentration in the medium. After careful consideration of both options, we decided to use the CoCl_2_ hypoxia model in our experiments, with the addition of a verification step involving the repetition of one of our experiments with 1% O_2_ hypoxia. We chose to study CREB phosphorylation as it is a signaling pathway and an early step in the process. In addition, it can be performed without opening the hypoxic incubator and without disrupting the hypoxic conditions. Similar to the results observed for MASP-1 and CoCl_2_, we found an interaction between 1% O_2_ hypoxia and MASP-1, which justifies the use of CoCl_2_ as a hypoxia model in our experiments.

HUVECs serve as a primary endothelial cell model, commonly used in the literature, and they can be readily prepared in large quantities. The heterogeneity of different endothelial cell lines is also well-known, raising the need for the usage of several biological replicates, for which HUVECs also serve as a suitable model. To ensure the broader applicability of our findings across diverse endothelial cell types, we replicated the CREB signaling pathway experiment using HUAECs, a primary arterial endothelial cell line. In contrast to HUVECs, preparing HUAECs freshly in a standardized manner poses challenges. Nonetheless, we successfully replicated the observed interaction of hypoxia and MASP-1 on CREB phosphorylation in HUAECs, which supports the robustness of our findings and enhances the relevance of our study to human arterial diseases.

**Figure 8:**
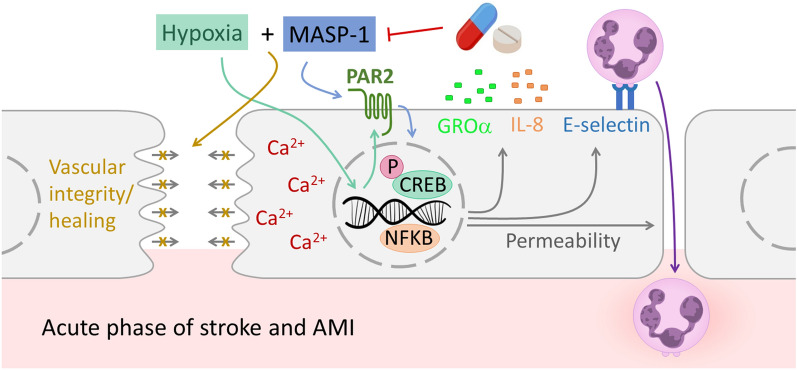
The proposed mechanism of the interaction of hypoxia and MASP-1 on endothelial cells Our results suggest that, through the enhanced expression of PARs, hypoxia potentiates the effect of MASP-1 on endothelial cells. This could possibly underlie the interaction observed at the level of signaling pathways (Ca^2+^, CREB, and NFκB), adhesion molecules (E-selectin), and vascular network integrity. The increased E-selectin expression potentially enhances neutrophil adhesion and infiltration, well-known in the acute phase of stroke and AMI, thereby contributing to secondary injury through their pro-inflammatory actions. This, coupled with compromised vascular network integrity and impaired vascular healing, exacerbates tissue damage and worsens the overall prognosis. Therefore, targeting MASP-1 medically could possibly mitigate these harmful effects and improve the outcome of atherosclerosis-related acute events. Note that the figure includes cases where either hypoxia or MASP-1 had an effect separately, as well as cases where they exhibited an interaction.

Our findings suggest that hypoxia potentiates the effect of MASP-1 on endothelial cells, at least partly by increasing the expression of PARs. This leads to the interaction observed at the level of signaling pathways (Ca^2+^, CREB, and NFκB), adhesion molecules (E-selectin) and capillary network integrity. The interaction of hypoxia and MASP-1, observed at several levels of the process, may facilitate the development of the strong neutrophil infiltration well-known in the acute phase of stroke and AMI, contributing to secondary injury. This, together with their capacity to impede vascular healing and cooperatively disrupt vascular network integrity, may exacerbate disease progression. Even with timely reperfusion therapy, there is still a significant mortality rate, which calls for further therapeutic options. Based on our finding that hypoxia and MASP-1 potentiate each other’s effects, we propose that MASP-1 can potentially be a drug target in the acute phase of atherosclerosis-related diseases (Fig. 8).

## Methods

### Reagents

The catalytic fragments of human recombinant MASP-1 (rMASP-1) were expressed in Escherichia coli as described by *Ambrus *et al*.*^57^ and refolded and purified according to *Dobó *et al*.*^58^ with slight modifications as described by *Megyeri *et al*.*^59^. The rMASP-1 construct contains the catalytic region of the molecule. It consists of the complement control protein domains 1 and 2 (CCP1, CCP2) and the serine protease domain (SP). The sequence begins with Gly298 and ends with Asn699. The CCP1-CCP2-SP catalytic fragment is enzymatically equivalent to the full-length molecule since it contains the catalytic domain and the CCP domains which provide exosites for the protein substrates. Specific enzymatic assessment of the rMASP-1 construct is described in the methods section of the Supplementary Material (see online). The recombinant enzyme fragments were endotoxin-free and could be inhibited by C1-inhibitor as described by *Megyeri *et al*.*^22^. Mouse anti-human E-selectin, Fluor568-conjugated goat anti-rabbit IgG, Streptavidin-Alexa488, SYBR Green and Fluo-4-AM were purchased from Invitrogen. Mouse anti-human ICAM-1 and ICAM-2 were purchased from Bender MedSystems. Mouse anti-human VCAM-1 was purchased from BD Pharmingen and rabbit anti-human HIF-1α was purchased from Abcam. Rabbit-anti-human phospho-CREB and rabbit-anti-human NFκB were purchased from Cell Signaling Technologies and Santa Cruz Biotechnology, respectively. Goat anti-mouse HRP was purchased from Southern Biotech. All other reagents were purchased from Sigma-Aldrich, unless otherwise stated.

### Preparation and culture of human umbilical vein endothelial cells (HUVECs) and human umbilical artery endothelial cells (HUAECs)

HUVECs and HUAECs were prepared from fresh umbilical cords of normally delivered healthy children by collagenase digestion as described by *Oroszlán *et al*.*^60^. For HUAEC isolation, the umbilical artery was dissected from the cord and collagenase was introduced through an 18G branula, while for HUVECs, the umbilical vein was kept intact in the cord and collagenase was administered using a glass cannula. Cells were cultured in gelatin-precoated flasks (Corning, Costar) in MCDB-131 medium (Life Technologies), completed with 5% heat-inactivated fetal calf serum (FCS) (PAN Biotech), 2  ng/ml human recombinant epidermal growth factor (hrEGF, R&D Systems), 1  ng/ml human recombinant basic fibroblast growth factor (hrBFGF), 0.3% Insulin Transferrin Selenium (Life Technologies), 1% Chemically Defined Lipid Concentrate (Life Technologies), 1% Glutamax (Life Technologies), 100 U/ml Penicillin and 100μg/ml Streptomycin antibiotics, 5  μg/ml Ascorbic acid, 250 nM Hydrocortisone, 10  mM HEPES and 7.5 U/ml Heparin (hereinafter referred to as Comp-MCDB). The cells were then cultured in vitro in Comp-MCDB medium. For the intracellular Ca^2+^ mobilization assay and the permeability tests, a modified version of AIM-V medium (Life Technologies) was used, completed with 1% filtrated, heat-inactivated FCS (PAN Biotech), 1  ng/ml hrBFGF growth factor, 2  ng/ml hrEGF (R&D Systems), and 7.5 U/ml Heparin (hereinafter: Comp-AIM-V). Hypoxic conditions were created by using 400 μM of CoCl_2_ or a 1% O_2_ hypoxic incubator. All experiments were performed on at least three independent primary HUVEC cultures from different individuals during passage 2 through 3. The CREB experiment was replicated using one HUAEC line at passage 12.

### Measurement of adhesion molecule expression by cellular ELISA and immunofluorescence microscopy

Confluent layers of HUVECs were cultured in 96-well plates for 24 h and then treated with 0.6 μM of rMASP-1, 400 μM of CoCl_2_ or both for 6 or 24 h. Alternatively, the cells were pretreated with 400 μM of CoCl_2_ for 2h and received 6h of 0.6 μM of rMASP-1 treatment after washing CoCl_2_ out. We used 100 ng/ml of LPS as a positive control. The cells were fixed in Methanol-Acetone for 10 min, then stained with mouse anti-human E-selectin, ICAM-1, ICAM-2, or VCAM-1 antibodies (1:500) for 1h at 37 °C. HRP-conjugated goat anti-mouse antibody was applied for 1h, and then 3,3’,5,5’-Tetra Methyl Benzidine (TMB) was utilized to develop color reaction. Alternatively, for E-selectin, Alexa Fluor568-conjugated goat anti-rabbit (1:500) IgG and Hoechst 33,342 (1:50000) were applied for 1h for fluorescent microscopic assessment. We examined E-selectin expression after 6h and ICAM-1, ICAM-2 and VCAM-1 expression after 24 h.

### Measurement of cytokine production by sandwich ELISA

Confluent layers of HUVECs were cultured in 96-well plates for 24 h, then treated with 0.6 μM of rMASP-1, 400 μM of CoCl_2_ or both for 24 h. IL-8 and GROα were measured by sandwich ELISA kits from the cell supernatant according to the manufacturer’s protocol (R&D Systems).

### Permeability assay

Permeability tests were performed using an XPerT technique^61^, slightly modified by *Debreczeni *et al*.*^24^. Confluent layers of HUVECs were cultured in 96-well plates pre-coated with 250 μg/ml of biotinylated gelatin for 2 days. After treatment with 400 μM of CoCl_2_ for 24 h and/or 0.6 μM of rMASP-1 for 20 min, 2 μg/ml of Streptavidin-Alexa488 was applied to each well for 2 min, and cells were fixed in 1% paraformaldehyde-PBS. We used 1 U/ml of thrombin as a positive control. Olympus IX-81 fluorescence microscope and an Olympus XM-10 camera were used to take pictures of each well. The plates were then read by a fluorescent plate reader (TECAN Infinite M1000 PRO) to determine the size of the stained area.

### Wound healing assay

HUVECs were cultured in 96-well plates until 100% confluency, then scratched. After one wash with Comp-MCDB medium, cells were treated with 0.6 μM of rMASP-1, 400 μM of CoCl_2_ or both. A combination of 5 ng/ml of bFGF and 10 ng/ml of EGF was used as a positive control. An Olympus CM30 Incubation Monitoring System was employed to capture images at 20 min intervals and analyze cell confluency.

### Capillary network integrity—Matrigel assay

HUVECs were seeded onto Matrigel^™^-coated 15-well Angiogenesis µ-Slides at 120% confluency. After 18 h of tube formation, cells were treated with 0.6 μM of rMASP-1, 400 μM of CoCl_2_, or both. Images were taken every 15 min with an Olympus CM30 Incubation Monitoring System. The number of branching points was measured using Image J software.

### Measurement of intracellular Ca^2+^ signaling by fluorescence microscopy

Intracellular Ca^2+^ mobilization was measured as described by our group earlier. Confluent layers of HUVECs were cultured in 96-well plates for 24h followed by pretreatment with 400 μM of CoCl_2_ for 4 h or 24 h. The cells were then loaded with 2 μM of Fluo-4-AM for 20 min, followed by a 20 min incubation in HBSS. Fluorescence microscopy was performed using an Olympus XM-10 camera. Baseline fluorescence was determined by taking three photos before adding 0.6 μM of rMASP-1, followed by sequential images every 5 s for 2 min. Changes in fluorescence intensity were calculated by analyzing at least 20 cells per image using CellP software. We used 50 μM of Histamine as a positive control.

### Measurement of CREB phosphorylation and NFκB nuclear translocation by immunofluorescence microscopy

For CREB experiments, HUVECs were treated with 0.6 μM of rMASP-1, 400 μM of CoCl_2_, or both for 2 h with or without 6 μM of C1INH. A second plate of HUVECs was placed in a hypoxic (1% O_2_) incubator with or without 0.6 μM of rMASP-1 treatment for 2 h. In another experimental setup, HUVECs/HUAECs were treated with varying concentrations of rMASP-1 (0.06 μM, 0.2 μM, 0.6 μM, 2 μM), 400 μM of CoCl_2_, or the combination of each dose of rMASP-1 with CoCl_2_ for 2h. For NFκB experiments, HUVECs were treated with 400 μM of CoCl_2_ for 2 h with/without 0.6 μM of rMASP-1 in the last 1h. We used 1 ng/ml of IL-1β as a positive control in both CREB and NFκB experiments. Cells were then fixed in Methanol-Acetone and stained with rabbit-anti-human phospho-CREB (pCREB, 1:200) or rabbit-anti-human NFκB (1:250) followed by Alexa Fluor568-conjugated goat anti-rabbit (1:500) IgG and Hoechst 33342 (1:50000). For NFκB experiments, additional Alexa Fluor568-conjugated wheat germ agglutinin (WGA, 1:200) staining was added after for 20 min. Olympus IX-81 fluorescence microscope and an Olympus XM-10 camera were used to take photos. Photos were analyzed using CellP software (Olympus). Nuclear mean red fluorescence (pCREB) or the difference between cytoplasmic and nuclear mean red fluorescence (NFκB) were calculated.

### Quanitative real-time PCR and mRNA purification

Confluent layers of HUVECs were cultured in 6-well plates and treated with 400 μM of CoCl_2_ for 30 min, 1, 3 or 6h. Total RNA was purified from cells using the illustra RNAspin Mini 50 Kit (GE Healthcare). RNA-cDNA transcription was performed using the Tetro cDNA Synthesis Kit Reverse Transcriptase (Bioline). The SensiFAST SYBR No-ROX Kit (Bioline) was used to quantify cDNA on a LightCycler (Roche). PAR1, 2 and 4 primers were designed from the NCBI Nucleotide database, and purchased from IDT (Coralville, IA). β-actin gene-specific primers were synthesized according to the published cDNA sequences (5-ATCAAGATCATTGCTCCTCCTGA-3 and 5-AAGGGTGTAACGCAACTAAGTCA-3). The sequences of the PAR1, 2 and 4 primers were as follows: 5-CTGTGTACACCGGAGTGTTTGT-3 and 5-AGTAAAATGCTGCAGTGACGAA-3 for PAR1 -AAGAGGGCCATCAAACTCATT-3 and 5-GTTCTTTGCATGATCCCTGAA-3 for PAR2 and -ACCATGCTGCTGATGAACCT-3 and 5-AGCACTGAGCCATACATGTGAC-3 for PAR4. The purity and the size of the PCR products were checked by melting curve analysis. Data were normalized with values of β-actin.

### Statistical analysis

The measurements were repeated on at least three independent HUVEC lines in at least two analytical replicates. Statistical analyses were performed after evaluating normality, using either nested one-way ANOVA with a Sidak multiple comparison post-test or one-way ANOVA with a post-test for linear trends or two-way ANOVA with a Sidak or Tukey’s multiple comparison post-test using GraphPad Prism 9.0 software. A p-value less than 0.05 was considered statistically significant. Data are presented as means ± SEM unless otherwise stated. In the figures, the level of significance is indicated only for the comparisons that are essential to demonstrate the interaction between hypoxia and MASP-1.

### Study approval

All experiments were performed in accordance with the WMA Declaration of Helsinki. All experimental protocols were approved by the Semmelweis University Institutional Review Board (permission number: TUKEB141/2015). All participants gave their written informed consent prior to inclusion.

### Supplementary Information

Supplementary Information 1.

Supplementary Video 1.

Supplementary Video 2.

## Data Availability

The datasets generated during and/or analyzed during the current study are available from the corresponding author on reasonable request.
