# Supplementary material for: Detrimental interactions of hypoxia and complement MASP-1 in endothelial cells as a model for atherosclerosis-related diseases
Source: Sci Rep. 2024 Jun 27;14:14882. doi: 10.1038/s41598-024-64479-6 (PMC11211410; doi:10.1038/s41598-024-64479-6)
Supplement: Supplementary file 1 — Supplementary Information 1. [file 41598_2024_64479_MOESM1_ESM.pdf]

Detrimental interactions of hypoxia and complement MASP-1 in endothelial cells as a model for atherosclerosis-related diseases

Flóra Demeter[1], Zsuzsanna Németh[1], Erika Kajdácsi[1,2], György Bihari[1], József Dobó[3], Péter Gál[3], László Cervenak[1]

[1] Research Laboratory, Department of Internal Medicine and Hematology, Semmelweis University, Budapest, Hungary

[2] Research Group for Immunology and Haematology, Semmelweis University— HUN-REN-SU (Office for Supported Research Groups), Budapest, Hungary

[3] Institute of Enzymology, HUN-REN Research Centre for Natural Sciences, Hungarian Research Network, Budapest, Hungary

SUPPLEMENTARY MATERIAL

Methods

Specific enzymatic activity assessment of the rMASP-1 construct

According to our measurements the catalytic efficiency of the MASP-1 construct on C2 substrate (k cat /K m = 3.0 x 10 5 M -1 s -1) [1] is in good agreement with the catalytic efficiency of the full-length molecule on the same substrate (k cat /K m = 3.4 x 10 5 M -1 s -1) [2]. We also measured the specificity constant of the recombinant MASP-1 construct on synthetic substrate (Z-Gly-Arg-S-Bzl) which resulted in 1.55 x 10 6 M -1 s -1 [3], which is quite reproducible for the different batches. The activity of rMASP-1 (CCP1-CCP2-SP fragment) was measured using D-Val-Leu-Arg p-nitroanilide as a substrate in 50 mM HEPES, 140 mM NaCl, pH 7.4, 0.1 mM EDTA, 0.1 % PEG-3350 buffer at 25 °C at 10 nM final enzyme and 200 μM final substrate concentration. P-nitroaniline was detected at 405 nm, and the reaction rates were calculated from the initial slope using the $\varepsilon = 9960\ M^{-1}cm^{-1}$ extinction coefficient. The specific activity of rMASP-1 ($v_0/[E]_T$) was $3.30 \pm 0.15\ s^{-1}$ under these conditions.

Visualization of HIF-1α nuclear translocation by immunofluorescence microscopy

Confluent layers of HUVECs were seeded onto 96-well plates, and incubated for 24h, before treatment with 100 or 400 μM of $CoCl_2$ for 2 or 24h. After 10 min of Methanol-Acetone fixation, rabbit anti-human HIF-1α (1:500), followed by Alexa Fluor®568-conjugated goat anti-rabbit (1:500) IgG and Hoechst 33342 (1:50000) were applied. Olympus IX-81 immunofluorescence microscope and an Olympus XM-10 camera were used to study HIF-1α nuclear translocation.

$CoCl_2$ cytotoxicity

Confluent layers of HUVECs were cultured in 96-well plates, then treated with 50/ 100 / 200 / 400 / 800 μM of $CoCl_2$ for 24h. We used 9% Dimethyl Sulfoxide (DMSO) as a positive control. Cell viability was scored based on confluency from 0 to 4 (0: 0%, 1: 25%, 2:50%, 3: 75%, 4:100%) at 1h, 2h, 4h and 24h by using light microscopy. At 24h, cells were fixed in Methanol-Acetone for 10 min, followed by a SYBR Green staining (1:5000). The plates were then read by a fluorescent plate reader (TECAN).

Results

Validation of $CoCl_2$ as a hypoxia model

1. HIF-1α nuclear translocation

As in most of our experiments, we used $CoCl_2$ instead of a hypoxic incubator to induce hypoxia, we first verified its hypoxia-mimetic properties by showing its time- and dose-dependent effect on HIF-1α nuclear translocation. Confluent layers of HUVECs were treated with 400 μM $CoCl_2$ for 2 or 24h. Fluorescence microscopy showed that 2 hours of $CoCl_2$ treatment significantly increased the HIF-1α concentration in the nuclei of HUVECs (**Supplementary Figures 1A and B**). This effect

became even more pronounced after 24h. Similar to time-dependence, we also investigated the dose-dependent effect of $CoCl_2$ on HIF-1α nuclear translocation. HUVECs were treated with 100 μM or 400 μM $CoCl_2$ for 24h. We found that the 400 μM $CoCl_2$ treatment increased the nuclear HIF-1α fluorescence intensity to a greater extent than the 100 μM $CoCl_2$ treatment (**Supplementary Figure 1C**).

2. Cytotoxic effect of $CoCl_2$ on ECs

After verifying the hypoxia-mimetic properties of $CoCl_2$, we investigated its potential cytotoxic effect on endothelial cells to determine the optimal concentration for our experiments. Confluent layers of HUVECs were treated with 50 / 100 / 200 / 400 μM $CoCl_2$ for 24h. The confluency of the cells was determined at 1h, 2h, 4h and 24h, by using light microscopy. On the basis of the confluency values, we found that, up to 400 μM concentrations, $CoCl_2$ did not exert any cytotoxic effect on endothelial cells, even at the latest time point (24h) (**Supplementary Figure 2A**). DMSO acted as a positive control. At 24h, cells were fixed and stained with SYBR Green fluorescent dye, and plates were read with a fluorescent plate reader. Fluorescence intensity measurements indicated that $CoCl_2$ did not have a cytotoxic effect on endothelial cells at 24h, even at the highest (400 μM) concentration (**Supplementary Figure 2B**). On the basis of these results, we used 400 μM $CoCl_2$ to induce hypoxia in our subsequent experiments.

Figures

Supplementary Figure 1

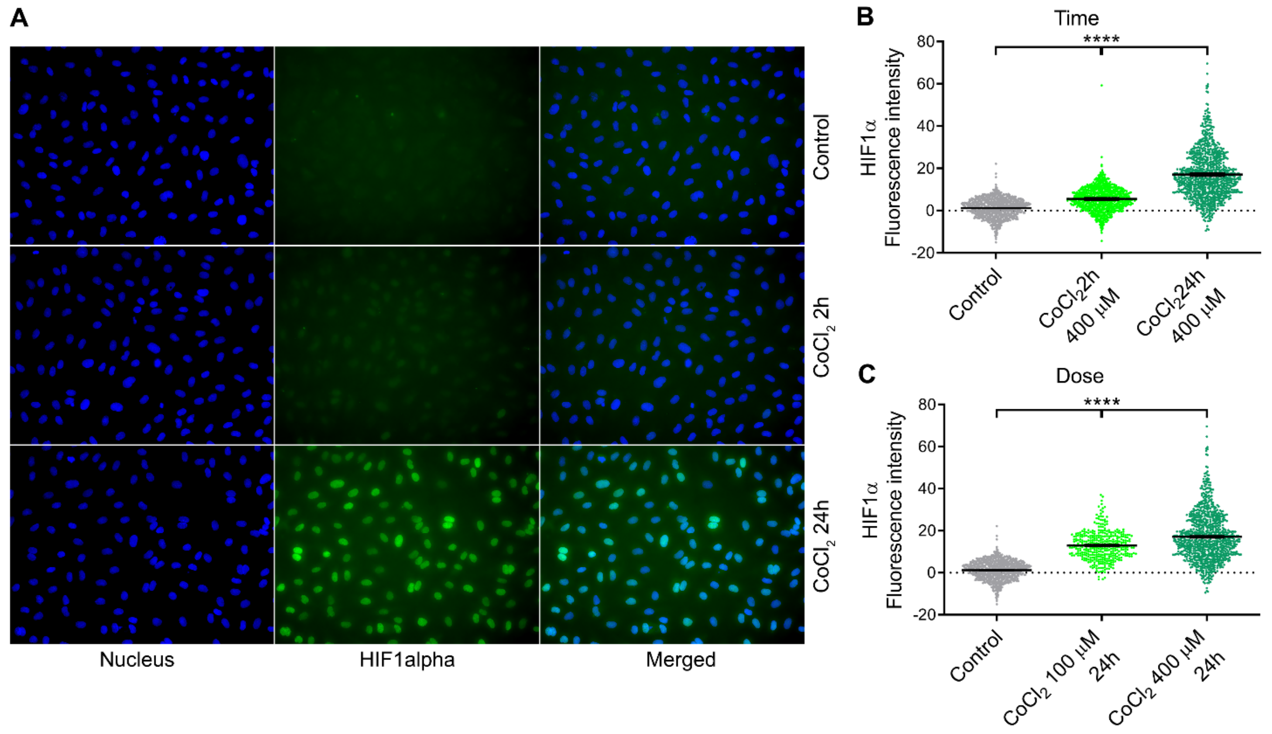

Supplementary Figure 1. **The effect of $CoCl_2$ on HIF-1α nuclear translocation**

Confluent layers of HUVECs were treated with 100 μM or 400 μM of $CoCl_2$ for 2 or 24h. After fixation, cells were stained with rabbit monoclonal anti-HIF-1α antibodies, followed by goat-anti-rabbit Alexa 568 (green) and Hoechst 33342 (blue) as nuclear staining. Images were obtained by using immunofluorescence microscopy (**A**). Panel **A** represents one set of photos from three independent experiments. To quantify the results seen in the fluorescent microscopic photos, image analysis was carried out using CellP software (**B, C**). n = 900 (3 biological replicates, 300 nuclei). Graphs represent individual values and mean ± SEM. One-way ANOVA with a post-test for linear trends. P values are as follows: ****: < 0.0001

Supplementary Figure 2

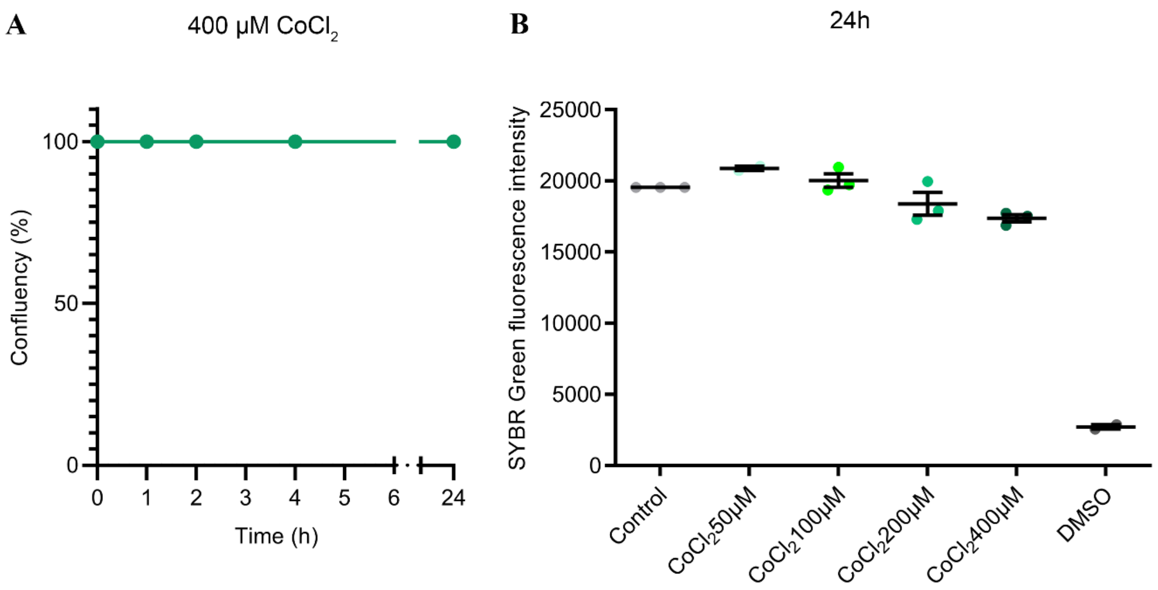

Supplementary Figure 2. The effect of $CoCl_2$ on the viability of ECs

Confluent layers of HUVECs were treated with 50 / 100 / 200 / 400 µM of $CoCl_2$ for 24h. The confluency of the cells was determined at 1h, 2h, 4h and 24h, by using light microscopy. Panel **A** shows the time-kinetic curve of cells treated with 400 µM of $CoCl_2$. At 24h, cells were fixed and stained with SYBR Green fluorescent dye. The fluorescence intensity of each well was determined with a fluorescent plate reader (**B**). n = 6-9 (2-3 biological replicates, 3 technical replicates). Graphs represent individual values and mean ± SEM. 9% Dimethyl Sulfoxide (DMSO) was used as a positive control. Nested one-way ANOVA with a Sidak multiple comparison post-test (nonsignificant).

Supplementary Figure 3

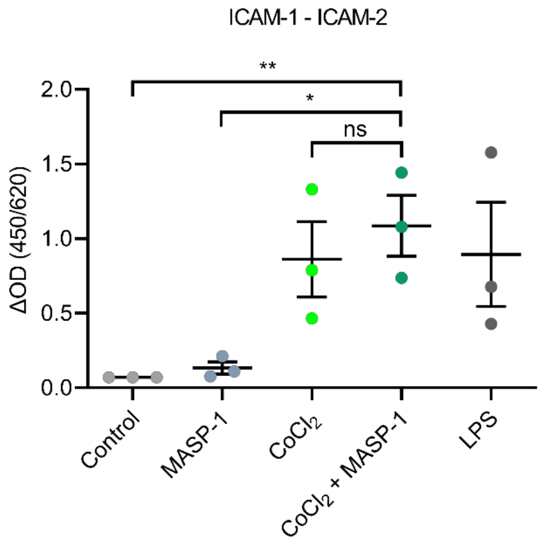

Supplementary Figure 3. The effect of $CoCl_2$ and rMASP-1 on the differential expression of ICAM-1 and ICAM-2

HUVECs were treated with 400 µM of $CoCl_2$ or 0.6 µM of rMASP-1 or both for 24h. We used 100 ng/ml of LPS as a positive control. After fixation, cells were stained with mouse anti-human ICAM-1 or ICAM-2 antibodies (1:500) for 1h followed by HRP-conjugated goat anti-mouse antibody and 3,3',5,5'-Tetra Methyl Benzidine (TMB) for 1h. Values were calculated by subtracting the mean ICAM-2 values from the mean ICAM-1 values. n = 3 biological replicates. The graph represents individual values and mean ± SEM. One-way ANOVA with a Sidak multiple comparison post-test. P values are as follows: *: 0.0141, **: 0.0099, ns: nonsignificant

Supplementary Figure 4

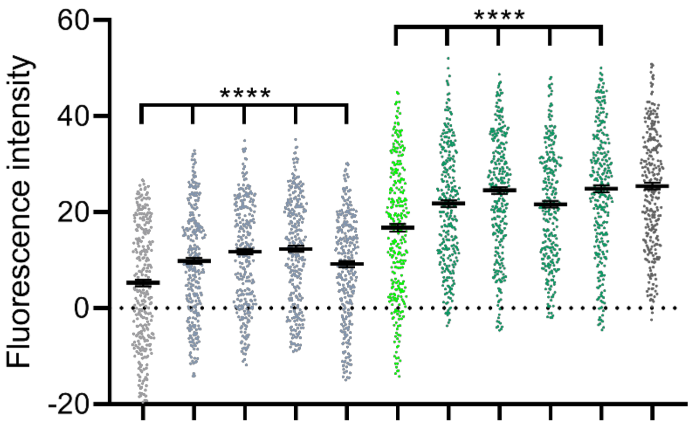

CoCl$_2$ (µM)	-	-	-	-	-	400	400	400	400	400	-
MASP-1 (µM)	-	0,06	0,2	0,6	2	-	0,06	0,2	0,6	2	-
IL-1β (ng/ml)	-	-	-	-	-	-	-	-	-	-	1

Supplementary Figure 4. Dose-response analysis of rMASP-1 on CREB phosphorylation

HUVECs were treated with varying concentrations of rMASP-1 (0.06 µM, 0.2 µM, 0.6 µM, 2 µM), 400 µM of $CoCl_2$, or the combination of each dose of rMASP-1 with $CoCl_2$ for 2h. We used 1 ng/ml of IL-1β as a positive control. After Methanol-acetone fixation, cells were stained with rabbit-anti-human phospho-CREB (1:200), followed by Alexa Fluor568-conjugated goat anti-rabbit (1:500, red) IgG and Hoechst 33342 (1:50000, blue). An Olympus IX-81 fluorescence microscope and an Olympus XM-10 camera were used to take photos. The nuclear mean red fluorescence was calculated after subtracting the cell-free background fluorescence values. n = 310 (1 biological replicate, 310 nuclei). Graphs represent individual values and mean ± SEM. One-way ANOVA with a post-test for linear trends. ****: $p < 0.0001$. Two-way ANOVA with a Sidak multiple comparison post-test was also conducted, comparing the combination of each MASP-1 dose with $CoCl_2$ to its respective MASP-1-only treatment pair (all p values < 0.0001); however, these results are not depicted on the figure for enhanced clarity.

Supplementary Video 1. The effect of $CoCl_2$ and rMASP-1 on wound healing

HUVECs were cultured in 96-well plates until reaching full confluency, when a scratch was created on the cell layer. After a single rinse with MCDB medium, cells were treated with 0.6 µM of rMASP-1, 400 µM of $CoCl_2$, or both. A combination of 5 ng/ml of bFGF and 10 ng/ml of EGF was used as a positive control. The Olympus CM30 Incubation Monitoring System was used to capture images at 20-minute intervals, which were subsequently converted into a video. The red lines indicate the initial border of the wound.

Supplementary Video 2. The effect of $CoCl_2$ and rMASP-1 on capillary network integrity

HUVECs were seeded onto Matrigel™-coated 15-well Angiogenesis µ-Slides at 120% confluency. After an 18-hour period of tube formation, cells were treated with either 0.6 µM of rMASP-1, 400 µM of $CoCl_2$, or a combination of both. Images were obtained with an Olympus CM30 Incubation Monitoring System and subsequently converted into a video. The red arrows point to a few examples of disappearing junctions, markers of the complexity of the capillary network.

Supplementary references

1 Ambrus, G. *et al.* Natural substrates and inhibitors of mannan-binding lectin-associated serine protease-1 and -2: a study on recombinant catalytic fragments. *Journal of immunology (Baltimore, Md. : 1950)* **170**, 1374-1382 (2003). https://doi.org:10.4049/jimmunol.170.3.1374

2 Chen, C. B. & Wallis, R. Two mechanisms for mannose-binding protein modulation of the activity of its associated serine proteases. *The Journal of biological chemistry* **279**, 26058-26065 (2004). https://doi.org:10.1074/jbc.M401318200

3 Megyeri, M. *et al.* Quantitative characterization of the activation steps of mannan-binding lectin (MBL)-associated serine proteases (MASPs) points to the central role of MASP-1 in the initiation of the complement lectin pathway. *The Journal of biological chemistry* **288**, 8922-8934 (2013). https://doi.org:10.1074/jbc.M112.446500
